# Optical monitoring of hemodialysis using noninvasive measurement of uric acid in the dialysate

**DOI:** 10.1038/s41598-023-40335-x

**Published:** 2023-08-17

**Authors:** Wojciech Żyłka, Krystyna Tęcza, Krzysztof Szemela, Piotr Prach, Marta Żyłka, Dorota Jakubczyk, Maciej Błądziński, Agnieszka Gala-Błądzińska, Paweł Jakubczyk

**Affiliations:** 1 Institute of Materials Engineering, College of Natural Sciences, University of Rzeszow, Rzeszow, Poland; 2 Institute of Medical Sciences, Medical College of Rzeszow University, Rzeszow, Poland; 3 Institute of Physics, College of Natural Sciences, University of Rzeszow, Rzeszow, Poland; 4 University of Information Technology and Management in Rzeszow, Rzeszow, Poland; 5 The Faculty of Mechanical Engineering and Aeronautics, Department of Aerospace Engineering, Rzeszow University of Technology, Rzeszow, Poland; 6 Faculty of Mathematics and Applied Physics, Rzeszow University of Technology, Rzeszow, Poland; 7 Department of Internal Medicine, Nephrology and Endocrinology, St. Queen Jadwiga Clinical District Hospital No. 2 in Rzeszow, Rzeszow, Poland

**Keywords:** Biophysics, Nephrology

## Abstract

The aim of this study was to present a methodology for predicting changes in uric acid concentrations in the blood of chronically hemodialyzed patients based on an optical measurement of the intensity of selected wavelengths in the dialysate. Blood samples were taken from the arterial line every 30 min throughout the hemodialysis period, to measure uric acid levels. Simultaneously, optical measurements were made on dialysate flowing from the dialyzer. Uric acid concentration can be measured either directly from the blood or from dialyzer outflow with acceptable error. In addition, both methods reveal any increased dynamics in uric acid concentration in the initial phase of hemodialysis. The wavelength of the light was adjusted for optimal uric acid particle detection. Comparing the uric acid concentration measured in the blood of patients with the intensity of wave absorption in the dialysate, the functional relationship between the uric acid concentration levels was determined. Using the optical method for measuring uric acid concentration in the dialysate, the concentration of uric acid in the blood during hemodialysis can be non-invasively and accurately estimated. This method can be used to assess the adequacy of hemodialysis by computer acquisition of uric acid concentrations determined in on-line dialysate.

## Introduction

Renal failure requiring renal replacement therapy affects many people in the world^1,2^. The incidence of cardiovascular complications, and the life expectancy and quality of life of chronically hemodialysed patients all largely depend on the optimal treatment of hemodialysis^3–5^. To assess the adequacy of hemodialysis (HD) during clinical practice, both clinical and laboratory parameters are evaluated. The degree to which uremic toxins are removed is one of the important components in assessing the adequacy of hemodialysis^6^. Measuring and assessing uremic toxin excretion is usually done by testing the patient’s blood once in the month before and after hemodialysis.

This assessment enables clinicians to predict subsequent hemodialysis treatment procedures, however it is an inaccurate assessment method.

Nowadays, the assessment of the adequacy of hemodialysis is based on, among others, the assessment of urea concentration in the blood serum before and after hemodialysis and the rate of its elimination from the body. It is calculated for this purpose Kt/V ratio determined in a one-compartment model^7^.

Uric acid is formed in the human body from the metabolism of purine bases^8^. In healthy kidneys, 70–75% of it is freely excreted in the urine^9^. In hemodialysis patients, hyperuricemia is found in 50% of patients on chronic hemodialysis^10^. Uric acid is a substance with a low molecular weight (168 Da), and is freely dialysed during the classical hemodialysis procedure, and removed with the used dialysate. Spectrophotometry is a method that has been described in medicine since the 1840s^11^.

The dynamics of changes in the concentrations of low-molecular-weight uremic toxins not bound to proteins in the blood is subject to kinetic modelling and can be approximated by exponential functions in single or multi-compartment models. Modelling of urea, which is a uremic toxin that is also an ineffective osmolyte (it easily penetrates between the water spaces of the body and in the way of diffusion between the blood compartment and the dialysis fluid compartment during haemodialysis), was developed and clinically confirmed many decades ago^12,13^.

Optical monitoring of uremic marker molecules in the used dialysate for online estimation and removal of uremic toxins, while determination of Kt/V based on the UV absorption of the used dialysate has been clinically validated and is used in clinical practice^14,15^.

The main of this study is to prove the hypothesis that the measurements of light absorption by a dialysate can be used to develop an a comfortable and no invasive method for estimating the uric acid concentration in patient's blood.

Three main system-based approaches to on-line monitoring of HD can be distinguished, electrochemical systems^16^, conductometric systems^17^ or the most popular optical systems, see for example^18–20^. In this work, we focus on the optical system approach, which exploits the fact that electromagnetic waves of specific wavelengths are absorbed when passing through uric acid of different concentrations, see for example^21^

The aim of the study was to assess the clinical usefulness of measuring uric acid concentration in dialysis fluid using a specially designed optic device, and then testing the validity of results by comparing with the levels of uric acid excretion determined in patients’ blood tests during hemodialysis.

## Materials and method

For our pilot study, we recruited 10 patients with end-stage renal disease undergoing hemodialysis. The reasons for kidney failure in the patients were: diabetic kidney disease (n = 4; 40%), hypertensive kidney disease (n = 2; 20%), chronic cardiorenal syndrome (n = 2; 20%), amyloidosis secondary to seronegative arthritis (n = 1; 10%), and autosomal dominant polycystic kidney disease (ADPKD) (n = 1; 10%).

All patients underwent hemodialysis (HD) on either the 1st or the 2nd of July 2019. Patients were treated with hemodialysis using a low-flux, single-use, hollow-fiber dialyzer with a POLYNEPHRON™ membrane. Dialyzers with an area of 1.7 or 1.9 or 2.1 m^2^ were used. In 7 (70%) patients HD was performed via an arterio-venous fistula in the wrist or elbow, and in the remaining 3 patients HD was performed via a tunneled catheter in the internal jugular vein. Three hemodialysis patients were given drugs during HD. One patient was given an intravenous infusion of iron (isomaltoside 1000 iron III) at a dose of 100 mg; one patient was given intravenous paricalcitol at a dose of 5 µg, at the beginning of their HD; and one patient was given intravenous erythropoietin (epoetin alfa) at a dose of 2000 IU, at the end of their HD.

This study was conducted in accordance with the principles of the Declaration of Helsinki and was approved by the Bioethics Committee of the University of Rzeszów (No. 9/05/2019).

Selected clinical and laboratory data as well as the results of measurements made during a single hemodialysis procedure in the examined patients are presented in Table 1.

**Table 1:** Characteristics of the patients according to selected features.

Patient	Sex	Age (years)	Height (m)	Weight (kg)		Diabetes	Time of HD (months)	BPs before HD (mmHg)	BPd before HD (mmHg)	BPs after HD	BPd after HD	UF (ml)	Effective blood flow (ml/min)	TMP
				Before HD	After HD									
D01-TA	M	67	1.76	92.5	90.2	1	19	117	79	112	65	2000	250	63
D02-MJ	M	61	1.68	98.2	95	1	31	162	78	150	81	3200	250	70
D05-GJ	F	81	1.57	79.8	78.5	1	8	153	78	133	66	1300	250	46
D06-TD	F	70	1.74	76.5	73.5	1	48	141	55	144	77	3300	250	72
D07-SA	F	75	1.56	52.5	51.5	0	46	125	56	175	83	1000	230	40
D08-ZZ	F	75	1.58	98.7	95.8	0	30	143	63	132	92	3000	250	73
D09-BS	F	65	1.78	–	–	0	3	133	58	124	75	1000	200	55
D10-PM	M	54	1.75	92.8	90	0	18	118	77	111	70	2900	220	64
D11-RF	F	79	1.47	83	81	0	36	151	85	101	55	2000	250	72
D12-MA	F	59	1.58	–	–	0	32	103	55	95	46	3000	210	105

*M* Male, *F* Female, *HD* hemodialysis, *BPd* diastolic blood pressure value, *BPs* systolic blood pressure value, *TMP* transmembrane pressure, *UF* ultrafiltration.

### Clinical measurement

In the studied group, uric acid was measured from blood taken during a single hemodialysis. Blood for testing was drawn from the blood supply line to the dialyzer during hemodialysis procedures according to the scheme: within the first 3 min from the start of HD and then every 30 min thereafter. The last blood sample was drawn at the end of the HD, just prior to the conclusion of the procedure. The blood serum tests for uric acid were performed in a hospital laboratory by an automated method with uricase and peroxidase on an Atellica® Solution analyzer immediately following the taking of each blood sample. The results of clinical measurement of uric acid for patients presented in Table 1 are presented in Table 2.

**Table 2:** Results of blood uric acid concentrations measurement.

Patient			D01-TA	D02-MJ	D05-GJ	D06-TD	D07-SA	D08-ZZ	D09-BS	D10-PM	D11-RF	D12-MA
Uric acid concentrations (mg/dl)	Time (min)	0	6.3	6.8	4.5	4.8	2.3	5.3	6.7	6	5.8	4.9
		30	2.2	2.2	2.6	3.1	1.6	4.2	4.6	4.8	3.7	3.5
		60	4.8	5.1	3	3.7	1.8	3.7	3.8	3.9	3	3
		90	4.2	4.3	2.5	2.6	1.3	3.6	3.1	3.4	2.5	2.5
		120	3.5	3.7	2.1	1.9	–	3.1	2.5	3	2.2	2
		150	3.1	3.2	1.9	1.6	1	2.6	2.2	2.8	1.9	1.8
		180	2.7	2.8	1.6	1.3	0.9	2.3	1.6	2.5	1.8	1.6
		210	–	2.6	1.5	–	–	2.1	–	–	–	–
		240	2.4	2.2	1.3	1.2	0.9	2	1.9	2.2	1.3	1.3

The unit of uric acid is (mg/dl).

**Figure 1:**
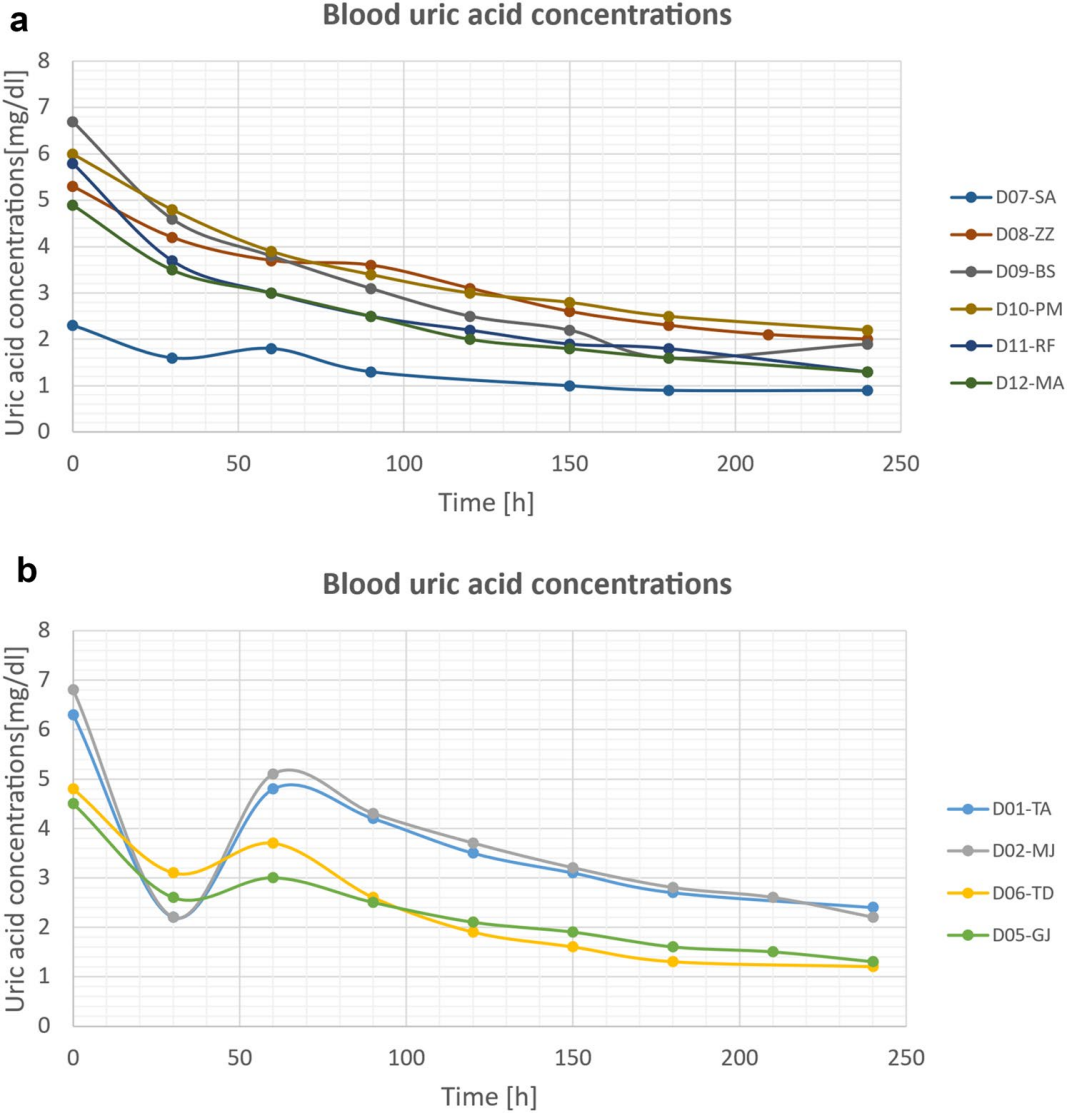
(**a**) Results of blood uric acid concentration measurements during a single hemodialysis procedure in patients without diabetes. (**b**) Results of blood uric acid concentration measurements during hemodialysis in patients with diabetes.

Due to the dynamics of changes in uric acid concentrations in the first phase of hemodialysis, the obtained results can be divided into two classes. The first class of results with lower dynamics corresponds to patients without diagnosed concomitant diseases, while the second class with noticeably higher dynamics of changes in uric acid concentrations corresponds to those diagnosed with diabetes. A graphic presentation of the results, divided into the two classes, is presented in the graphs in Fig. 1a and b.

### Optical measurement

For optical measurement, in our study we propose the use of a non-invasive dialysate drainage device (connected to the hemodialysis process) that measures uric acid concentration by spectrophotometry. For this purpose the absorption phenomenon of the light by a uric acid flowing through a transparent cable was employed. A photodetector placed on the opposite site of the cable can measure amount of transmitted light described by the transmission coefficient^22–25^. The absorption depends on the wavelength, hence, to simplify the analysis of the process of the light transmission, a monohromatic electromagnetic wave was used. An accuracy of the proposed method as well as its correctness and reliability strongly depends on the wavelength of the light used in the measurements. A uric acid should absorb the chosen monochromatic light in a proper amount. It is obvious that too small as well as too high absorption makes our sensor not sensitivity enough or even blind. Therefore, it is crucial to find an proper for our measurements length of the light. After choosing an appropriate wavelength, the next step is expressing a uric acid concentration as a simple mathematical function of this transmission coefficient.

### Determining a proper wavelength

To better know the phenomenon of the light absorption by a uric acid, the influence of the wavelength on the light transmission was experimentally investigated. The light absorption by some uric acid probes of different concentrations was examined. Based on the experimental data, the transmitted coefficient was presented in Fig. 3 as a function of the wavelengt. Figure 3 shows that there is a value of the wavelength for which the light absorption achieves its maximum for all the analyzed uric acid probes. To precisely determine this wavelength value, the dependence of the light absorption on the wavelength was approximated by means of the following polynomial: 0.0000000907λ^6^ – 0.0003276103λ^5^ + 0.4928330339λ^4^ – 395.1139625939λ^3^ + 178,048.12236675λ^2^ – 42757228.1737871λ + 4,274,817,397.85099. The polynomial approximation indicates that the light absorption achieves its maximal value for λ = 573 nm. This wavelength value ensure that the light absorption is not too small which makes our sensor sensitive. Therefore, the proposed device uses a laser emitting the light of this wavelength and the photodetector sensitive to this wavelength.

### Description of the measurement device

Measurements were made with a high performance 0.065 nm HR2000 fiber optic spectrometer. The HR2000 is sensitive in the range of 200–1100 nm, but to be precise, the range and resolution depend on the gratings and input slots selected. In Table 3 we present the specifications of the CCD detector in the HR2000 CCD detector, and of the HR2000 spectrometer itself. The most important parameters for testing are: detector range: 200–1100 nm; entrance aperture: 5, 10, 25, 50, 100 or 200 µm wide slits or fiber (no slit); optical resolution: depends on grating and size of entrance aperture and core diameter of 1.7 µm; dynamic range: 2 × 10^8^ (system); 2000:1 for a single scan; siber optic connector: SMA 905 to single-strand optical fiber (0.22NA).

**Table 3:** Results of optical measurement of intensities for the wavelength λ = 573 nm in spent dialysate.

Patients	Intensity (a.u.)												
	Time (min)												
	0	15	30	45	60	75	90	105	120	135	150	165	180
D01-TA	532	730	997	994	702	732	763	844	800	891	925	933	892
D02-MJ	474	489	980	990	690	710	780	795	893	920	976	993	1004
D03-DA	699	676	924	–	824	926	933	937	1037	–	1039	1073	–
D05-GJ	566	573	813	851	896	896	887	886	894	1036	1083	1089	1081
D06-TD	754	857	907	904	908	911	960	1028	1082	1082	1089	–	–
D08-ZZ	660	714	763	813	855	896	903	936	923	931	–	903	1083
D09-BS	469	635	717	762	846	893	923	907	960	957	1082	–	–
D10-PM	–	699	718	750	819	858	906	937	924	931	907	959	1024
D11-RF	655	732	780	856	904	923	931	950	950	953	1074	1082	1089
D12-MA	636	636	705	728	762	858	937	924	931	970	1024	1082	–

The diode that was used for the device is a diode emitting in the spectrum of 465–640 nm controlled with a voltage in the range from 3.4 to 3.7 V. Power 3 W, current 700 mA and angle 120°.

Applying the “brute force” method we empirically chosen the wavelength of the light equal to 573 nm as optimal for uric acid detection (see also^26,27^).

**Figure 2:**
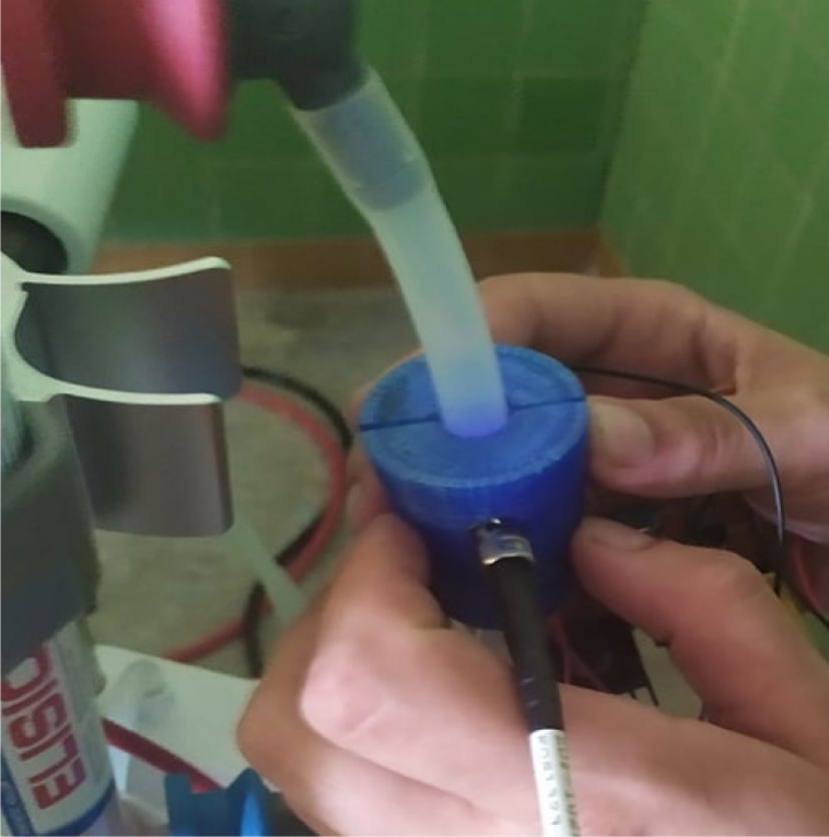
Photograph of the measuring point assembly.

Such light emitted by the device’s diode was passed through a transparent tube with flowing dialysate in a sealed casing, which prevents light from outside interfering with the measurements; see Fig. 2.

This sealed casing assembly is necessary to get test results that are without interference or noise. To include the influence of light scattering on the silicon tube, the optical measuring system was calibrated to take into account the opacity of the tube. This calibration was taken into consideration during calculating the intensities of the light entering the spectrometer. Uric acid contained in the dialysis fluid absorbs the incident light, and data are collected by a fiber optic cable leading to the spectrometer. The measurement and data acquisition time lasts 1 s. “Ocean Opitcs Spectra Suite” software was used to acquire data, create calculations, undertake mathematical analysis, and create a results chart.

The proposed method assumes that the measurements can be performed by using the “Intensity Measurement” function. The intensity is the power of the light entering the spectrometer and is measured by a photodetector placed on the opposite side of the tube through which the dialysate flows. Taking into account that the device is not calibrated, only related changes of the intensity can be measured. However, this fact is not important for our method. The main aim of the analysis is to find a simple mathematical function which expresses the uric acid concentration by the intensity value. Generally, it can be assumed that at this stage the intensity is measured in arbitrary units. It should be noted that such mathematical function depends on the device construction as well as on some optical properties of its components. Thus, this mathematical relation must be found for each device separately and this process can be considered as an initial calibration.

The device was operated on the “Absorbance Measurement” function, this means that it measures the amount of light reaching the spectrometer. The use of the abovementioned chemometric application is necessary because the device is calibrated to be used in conjunction with that software. Then the spectrometer creates a graph of the absorption of emitted light with an accuracy of 1 nm. A graph of each measurement and a graph of the absorption level of all measurements for a given patient over time were created.

After selecting the appropriate device components and laboratory tests, clinical trials began. The first measurement was taken when dialysis began, other measurements were taken every 15 min. In parallel, the medical team took blood samples every 30 min. To synchronize the measurement process, the first invasive and non-invasive measurements were taken at the same time.

### Measurement data processing

**Figure 3:**
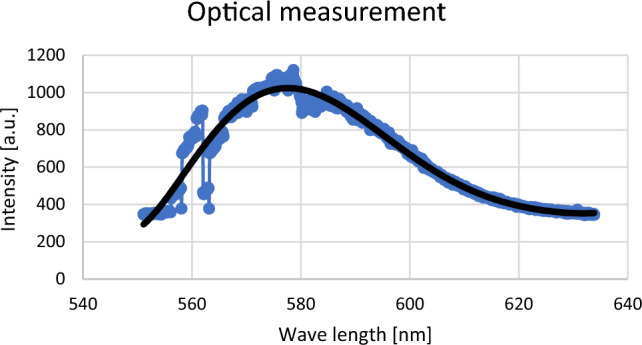
An example of the approximation of peak intensities during the measurement. Here the approximation polynomial has the form: y = 0.0000000907x^6^ − 0.0003276103x^5^ + 0.4928330339x^4^ − 395.1139625939x^3^ + 178,048.12236675x^2^ − 42,757,228.1737871x + 4,274,817,397.85099 with a determination coefficient of R^2^ = 0.9518918063.

In order to find a general method of calculating the intensity (in arbitrary units [a.u.]) of light absorbed by the detector in the spectrometer for a specific wave length λ, we have made a polynomial approximation of the intensities of peak positions (see an example of a single measured point presented in Fig. 3) for every measurement.

**Figure 4:**
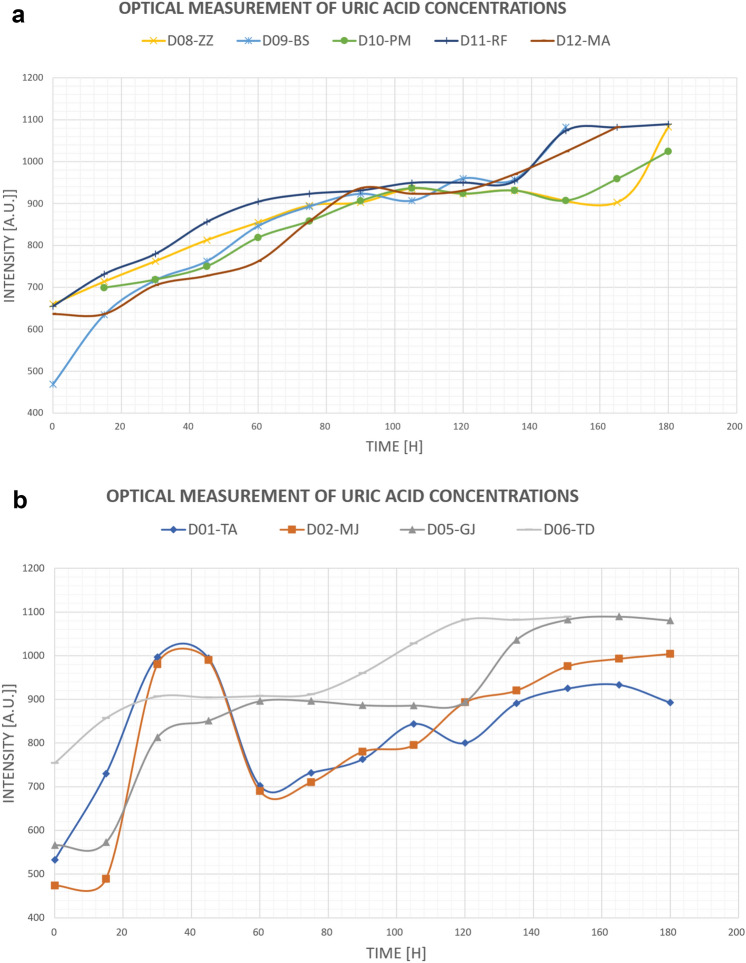
(**a**) Graph presentation of optical measurement of uric acid concentration in spent dialysate during a single hemodialysis procedure in patients without diabetes. (**b**) Graph presentation of optical measurement of uric acid concentration in spent dialysate measurements during hemodialysis in patients with diabetes.

Next, we calculated the value of the light intensity in terms of an approximating polynomial for the wavelength $\lambda =573 \,  {\text{nm}}$ which is optimal for uric acid particles detection. For example, in the case presented in Fig. 4, the measured value was calculated as $y\left(573\right)=996.74 \, {\text{a.u.}}$ The same procedure was applied to every measurement point. This measurement methodology guarantees that the intensity values obtained are optimal, and it gives the most accurate values even if there are interferences during the measurements. The results of optical measurements during hemodialysis for the wavelength $\lambda =573 \, {\text{nm}}$ which correspond to the detection of uric acid are presented in Table 3.

The data shown in Fig. 4a and b in order to distinguish patients without diabetes from patients with diabetes (cf. results presented in Fig. 1a,b).

**Figure 5:**
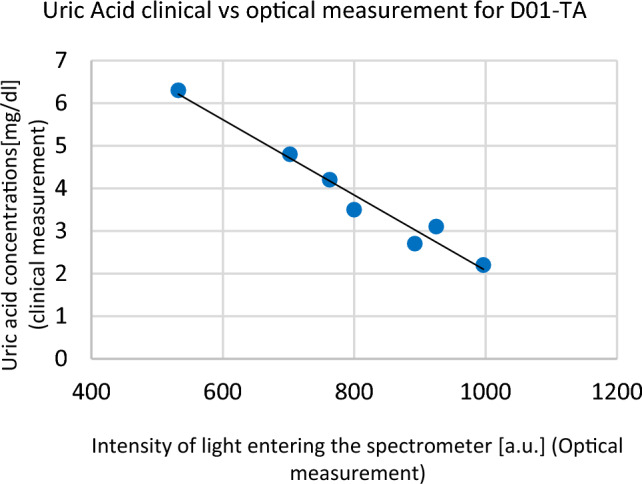
Linear dependence between the intensity of light entering the spectrometer and clinical measurement for uric acid assessed in patient D01-TA.

Figure 4a shows that the intensity increases during HD treatment. This behaviour can be explained by the fact that during hemodialysis uremic toxins are eliminated from the blood as well as from the dialysate. Decreasing the uric acid concentration causes an decreasing light absorption, and consequently leads to increase in the light intensity. In the case of some patients. The behaviour of the intensity in the case of some diabetic patients is more complicated (see Fig. 5b) which can be due to more complicate changes of uremic toxins in the blood during hemodialysis.

### Statement

Statement confirming that informed consent was obtained from all participants and/or their legal guardians.

## Comparison of clinical and optical measurement

Optical measurement was performed with the wavelength $\lambda =573$ nm to obtained results for the intensity of light which passes through the spent dialysate and enter to the spectrometer. Simultaneously clinical measurements were taken from blood samples every 30 min. We observed, that for all patients, there is a linear dependence between the clinical measurements of uric acid and the intensity of light entering the spectrometer (optical measurement). An example of a direct comparison of clinical uric acid concentrations and optical measurements of light entering the spectrometer for patient D01-TA at time points t = {0, 30, 60, 90, 120, 150, 180 min.} is shown in the Fig. 5.

As can be seen from Fig. 5, the concentration of uric acid decreases during HD, hence the amount of light reaching the spectrometer increases. This dependence was determined using the method of least squares and has the form $CM=-0.0088*OM + 10.919$ with a coefficient of determination of $R^{2} = 0.9676$. Here $CM$ stands for clinical measurement and $OM$ for optical measurement. Similar linear dependences can be observed for the remaining 9 patients (see Fig. 7), and across all patients, it takes the average form

1 $$CM=-0.008*OM + 10.427$$

with $R^{2} = 0.897$. The value of coefficient R is close to unity which indicates clearly that the uric acid concentration can be considered as a linear function of absorption measured by the optical device.

Equation (1) allows us to estimate the uric acid concentration in the blood, for all patients, in terms of the optical measurement of light entering the spectrometer. This crucial dependence is necessary to build a complete on-line measurement system of uric acid concentration during hemodialysis session.

**Figure 6:**
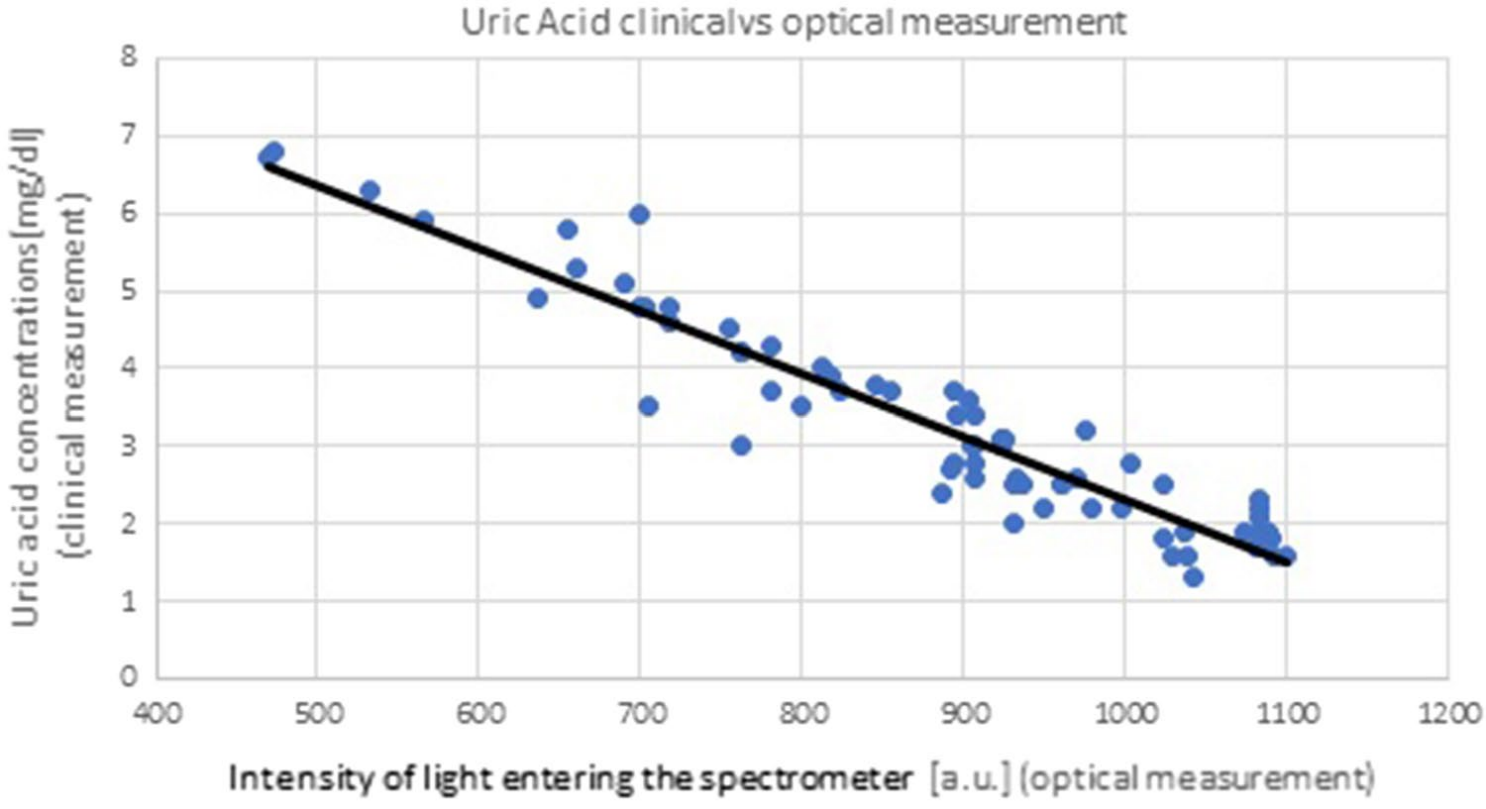
Linear dependence between the intensity of light entering the spectrometer and clinical measurements for uric acid performed for all patients.

**Figure 7:**
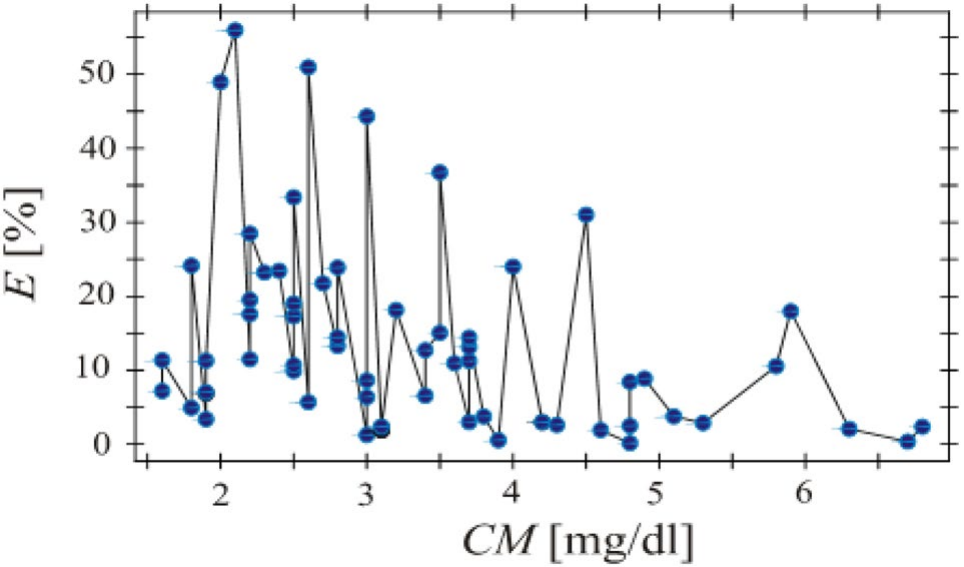
Relative error *E* as a function of uric acid concentration for all the dialysate samples.

The correctness of the formula from Eq. (1) was tested using the measurement results collected in Tables 2 and 3. Based on the optical measurements and with the use of the relation (1) the uric acid concentration in blood were estimated. Then, these values were compared with the values determined based on the patient's blood test. The analysis was performed for 64 dialysate samples. The following relative error

2 $$E=\frac{\left|CM-{CM}^{(op.)}\right|}{CM}\cdot 100\%$$

was defined where ${CM}^{(op.)}$ is the value of uric acid in patient's blood predicted using the formula (1). The relative error *E* was calculated for all the 64 pairs $\left(OM,CM\right)$ from Tables 2 and 3 and presented in Fig. 7. The quantity *E* as a function of the uric acid concentration was presented in Fig. 6.

Figure 7 shows that the relative error *E* assumes the value smaller than 11% when *CM* > 4.6 mg/dl with the exception of the single sample for which *CM* = 5.9 mg/dl. It can be noted that for low values of the uric acid concentration *CM* < 4.6 mg/dl, the optical measurements are not sufficiently accurate. This can be due to an optical measurement inaccuracy. Hence, it can be concluded that the prototype device has to be improved and investigations should be performed for greater number of dialysate samples. However, it can be stated that the proposed method can be useful for the non-invasive estimating uric acid in patient's blood.

## Discussion

In patients with terminal renal failure who are chronically hemodialyzed, various compounds called uremic toxins accumulate in the body between dialysis treatments. During hemodialysis, uremic toxins are removed simultaneously by at least two mechanisms: diffusion and ultrafiltration. The share of individual elements depends on the type of dialyzer membrane, its ultrafiltration coefficient, permeability coefficient and the molecular weight of the substance removed.

The uremic toxin that we decided to study, whose concentration in blood and dialysate occurs during hemodialysis, is uric acid. We selected this uremic toxin knowing that it is a small molecule that frees itself during hemodialysis. In addition, according to the latest research, blood uric acid concentration is a biomarker of mortality among chronically hemodialysis patients^28,29^.

In some studies, in recent years, it has been shown that uric acid behaves very similarly to urea during hemodialysis^18^.

In our study, we observed that the HR2000 optical spectrometer we used, measures uric acid concentration in dialysate with a good correlation to results from blood samples that were simultaneously tested in hemodialysis patients. The great advantage of the optical approach is its ability to measure uric acid in the dialysate very frequently, even every few minutes, which thus allows the clinician almost continuously control the hemodialysis process and trouble-free computer data acquisition, without having to take blood from the patient.

Other authors also evaluated the use of photometry in assessing the dynamics of uremic toxin removal using spectrophotometric measurement of the dialysate during the hemodialysis process^30,31^. In one of these studies^32^, the optimal wavelength for measuring uric acid concentration was indicated as $\lambda =297 \, {\text{ [nm]}}$ which obtained a correlation coefficient at the level of ${R}^{2}=0.7386$. In our work, we suggest using a wavelength approximately twofold, which gives a correlation coefficient of ${R}^{2}= 0.897$. It seems, therefore, that this approximately twofold increase in wavelength results in a better capture of uric acid molecules.

The functional relationship we found between uric acid concentration in dialysate and blood provides a fully non-invasive and non-disruptive method of estimating uric acid concentration in blood by examining the dialysate flowing into the sink.

In our study, we confirmed a good correlation (at least 90%) between uric acid concentrations as measured by dialysate spectrophotometry and uric acid concentrations as measured in a certified blood laboratory. In addition, by measuring both blood and dialysate, we observed a significant increase in the rate of uric acid excretion at the initial stage of hemodialysis in patients with underlying diabetes.

To the best of our knowledge the anomalous dynamics of uric acid concentration in diabetes patients in Fig. 1b (a relatively sharp drop at 30 min and subsequent return to typical exponential curves) has never been reported before and if reproduced is an interesting and original research result. The possible nature of this phenomenon may be related to the refiling that occurs during hemodialysis. During hemodialysis, fluid is removed from the patient’s vascular system by ultrafiltration. The reduction in intravascular fluid by ultrafiltration results in a compensatory fluid refill from the extravascular water space (interstitium, cells) into the intravascular water space^33^. de los Reyes et al.^34^ published a study describing the fluid dynamics of patients undergoing hemodialysis with ultrafiltration for one hour after the start of hemodialysis using a two-compartment model. In our study the steeper decline blood uric acid concentration in the first 30 min of hemodialysis diabetic patients is due to efficacious removal by dialysis, which is not matched by the effective refiling water-soluble toxins such uric acid in the extravascular compartment. Less efficient refilling in patients with diabetes mellitus may result from changes of a micro- and macrovascular nature accompanying dialysis patients with diabetes. This hypothesis of refiling disorders in the group of diabetic patients is confirmed by the known complication of more frequent intradialytic hypotension in diabetic patients compared to non-diabetic patients^35^. In our opinion the different dynamics of uric acid concentration in diabetic patients (relatively rapid decrease after 30 min and subsequent return to typical exponential curves) is an interesting and original research result requiring validation in a larger population of patients.

## Conclusions

The appropriate wavelength for measurements of a light absorption by a dialysate was determined. The prototype device was built and the method for predictions uric acid in blood based on optical measurements was proposed. The performed investigations show the linear dependence between uric acid concentration in blood and the absorption coefficient. This fact was used to not invasively estimate uric acid concentration in blood. The relative error of the predicted value uric acid in blood was investigated. The relative error can achieve some great values, especially when the concentration is low, which is due to measurements errors as well as imperfection of the prototype device. The hypothesis that measurements of light absorption by dialysate can be used to not invasively estimated uric acid concentration in blood was confirmed.

The use of photospectrometry as a method for assessing uric acid concentrations in dialysate during hemodialysis correlates with the uric acid concentrations found in patients’ blood sampled during hemodialysis, therefore it may be clinically useful as a non-invasive assessment of hemodialysis. For optical measurements, it appears that doubling the wavelength increases the efficiency of uric acid concentration estimation during hemodialysis.

## Data Availability

The datasets used and/or analyzed during the current study available from the corresponding author on reasonable request.

## References

[1] Liyanage T (2015). Worldwide access to treatment for end-stage kidney disease: A systematic review. Lancet.

[2] Bello AK (2019). Status of care for end stage kidney disease in countries and regions worldwide: International cross sectional survey. BMJ.

[3] Ahmadmehrabi S, Tang WHW (2018). Hemodialysis-induced cardiovascular disease. Semin. Dial..

[4] Rhee CM, Chou JA, Kalantar-Zadeh K (2018). Dialysis prescription and sudden death. Semin. Nephrol..

[5] Kraus MA (2016). Intensive hemodialysis and health-related quality of life. Am. J. Kidney Dis..

[6] Chan CT (2019). Conference participants. Dialysis initiation, modality choice, access, and prescription: Conclusions from a kidney disease: Improving global outcomes (KDIGO) controversies conference. Kidney Int..

[7] KDOQI Clinical Practice Guideline for Hemodialysis Adequacy (2015). 2015 update. Am. J. Kidney Dis..

[8] Hediger MA, Johnson RJ, Miyazaki H, Endou H (2005). Molecular physiology of urate transport. Physiology.

[9] Bobulescu IA, Moe OW (2012). Renal transport of uric acid: Evolving concepts and uncertainties. Adv. Chronic Kidney Dis..

[10] Suliman ME (2006). J-shaped mortality relationship for uric acid in CKD. Am. J. Kidney Dis..

[11] Toida T, Sato Y, Komatsu H, Kitamura K, Fujimoto S (2019). Pre- and postdialysis uric acid difference and risk of long-term all-cause and cardiovascular mortalities in Japanese hemodialysis patients. Miyazaki dialysis cohort study. Blood Purif..

[12] Schneditz D, Fariyike B, Osheroff R, Levin NW (1995). Is intercompartmental urea clearance during hemodialysis a perfusion term? A comparison of two pool urea kinetic models. J. Am. Soc. Nephrol..

[13] Burgelman M, Vanholder R, Fostier H, Ringoir S (1997). Estimation of parameters in a two-pool urea kinetic model for hemodialysis. Med. Eng. Phys..

[14] Castellarnau A, Werner M, Günthner R, Jakob M (2010). Real-time Kt/V determination by ultraviolet absorbance in spent dialysate: technique validation. Kidney Int..

[15] Petitclerc T, Ridel C (2021). Routine online assessment of dialysis dose: Ionic dialysance or UV-absorbance monitoring?. Semin. Dial..

[16] Keshaviah PR, Ebben JP, Emersonm PF (1995). On line monitoring of the delivery of the hemodialysis prescription. Pediatr. Nephrol..

[17] Aslam S, Saggi SJ, Salifu M, Kossmannm RJ (2018). Online measurement of hemodialysis adequacy using effective ionic dialysance of sodium: A review of its principles, applications, benefits, and risks. Hemodial. Int..

[18] Zemchenkov GA (2022). An optoelectronic spectral sensor for monitoring the elimination of uremic markers with low and middle molecular weight during hemodialysis therapy. Biomed. Eng..

[19] Vasilevsky AM (2015). Dual wavelength optoelectronic sensor for monitoring uric acid concentration in dialysate. Biomed. Eng..

[20] Konoplev GA, Stepanova OS, Zemchenkov GA, Frorip A (2019). Optical spectral sensor for the assessment of uric acid kinetics during hemodialysis treatment. J. Phys. Conf. Ser..

[21] Rashid NCA (2019). Spectrophotometer with enhanced sensitivity for uric acid detection. Chin. Opt. Lett..

[22] Walker JT (1949). The applications of spectrophotometry to medicine. N. Engl. J. Med..

[23] Fridolin I, Magnusson M, Lindberg LG (2001). Measurement of solutes in dialysate using UV absorption. Opt. Diagn. Sens. Biol. Fluids Glucose Cholest. Monit..

[24] Uhlin F (2003). Estimation of delivered dialysis dose by on-line monitoring of the ultraviolet absorbance in the spent dialysate. Am. J. Kidney Dis..

[25] Castellarnau A (2010). Real-time Kt/V determination by ultraviolet absorbance in spent dialysate: Technique validation. Kidney Int..

[26] Yaacob A, Ngajikin NH, Rashid NCA, Ali SHA, Yaacob M, Isaak S, Cholan NA (2020). Uric acid detection in visible spectrum. Telkomnika Telecommun. Comput. Electron. Control.

[27] Yaacob A, Ngajikin NH, Rashid NCA, Yaacob M, Alim SA, Azmi NE, Cholan N (2022). Linearity range enhancement in direct detection of low concentration uric acid. Optik.

[28] Sharma MK, Wieringa FP, Frijns AJH, Kooman JP (2016). On-line monitoring of electrolytes in hemodialysis: On the road towards individualizing treatment. Expert Rev. Med. Dev..

[29] Latif W (2011). Uric acid levels and all-cause and cardiovascular mortality in the hemodialysis population. Clin. J. Am. Soc. Nephrol..

[30] Ross EA, Paugh-Miller JL, Nappo RW (2018). Interventions to improve hemodialysis adequacy: Protocols based on real-time monitoring of dialysate solute clearance. Clin. Kidney J..

[31] Fridolin I, Magnusson M, Lindberg LG (2002). On-line monitoring of solutes in dialysate using absorption of ultraviolet radiation: Technique description. Int. J. Artif. Organs.

[32] Azar AT (2013). Modeling and Control of Dialysis Systems.

[33] Pietribiasi M, Waniewski J, Załuska A, Załuska W, Lindholm B (2016). Modelling transcapillary transport of fluid and proteins in hemodialysis patients. PLoS ONE.

[34] de los Reyes VAA, Fuertinger DH, Kappel F, Meyring-Wösten A, Thijssen S, Kotanko PA (2016). Physiologically based model of vascular refilling during ultrafiltration in hemodialysis. J. Theor. Biol..

[35] Li WH, Yin YM, Chen H, Rui ZR, Yuan Y, Yun H, Wang JW (2020). Clinical research on individualized hemodialysis preventing unconventional hypotension in diabetic nephropathy patient. Int. J. Artif. Organs..

